# IMPACT OF DEFICIENT NUTRITION IN BONE MASS AFTER BARIATRIC SURGERY

**DOI:** 10.1590/0102-6720201600010010

**Published:** 2016

**Authors:** Tatiana Munhoz da Rocha Lemos COSTA, Mariana PAGANOTO, Rosana Bento RADOMINSKI, Victoria Zeghbi Cochenski BORBA

**Affiliations:** Endocrinology and Metabolism Service, Hospital de Clínicas, Federal University of Paraná, Curitiba, PR, Brazil

**Keywords:** Bariatric surgery, Bone mineral density, Nutrients deficiency, Supplementation, Sun exposure

## Abstract

**Background:**

Essential nutrients are considered for the prevention of the bone loss that occurs after bariatric surgery.

**Aim:**

Evaluate nutrients involved in bone metabolism, and relate to serum concentrations of calcium, vitamin D, and parathyroid hormone, and the use of supplements and sun exposure on the bone mass of patients who had undergone gastric bypass surgery.

**Methods:**

An observational study, with patients who had undergone the surgery 12 or more months previously, operated group (OG), compared to a control group (CG).

**Results:**

Were included 56 in OG and 27 in the CG. The mean age was 36.4±8.5 years. The individuals in the OG, compared to CG, consumed inadequate amounts of protein and daily calcium. The OG had a higher prevalence of low sun exposure, lower levels of 25OH Vitamin D (21.3±10.9 vs. 32.1±11.8 ng/dl), and increased serum levels of parathyroid hormone (68.1±32.9 vs. 39.9±11.9 pg/ml, p<0.001). Secondary hyperparathyroidism was present only in the OG (41.7%). The mean lumbar spine bone mineral density was lower in the OG. Four individuals from the OG had low bone mineral density for chronological age, and no one from the CG.

**Conclusion:**

The dietary components that affect bone mass in patients undergoing bariatric surgery were inadequate. The supplementation was insufficient and the sun exposure was low. These changes were accompanied by secondary hyperparathyroidism and a high prevalence of low bone mass in lumbar spine in these subjects.

## Abstract (pt)

**Racional:**

Alguns nutrientes são essenciais para a prevenção da perda de massa óssea que ocorre após a cirurgia bariátrica.

**Objetivo:**

Avaliar nos pacientes que foram submetidos à cirurgia bariárica pela técnica de bypass gástrico os nutrientes envolvidos no metabolismo ósseo e sua relação com: a concentração sérica de cálcio; a vitamina D e paratormônio; o uso de suplementos alimentares e a exposição solar.

**Métodos:**

Estudo observacional com pacientes que foram previamente submetidos à cirurgia, 12 meses ou mais que compuseram o grupo operado (OG), em comparação a um grupo controle (GC).

**Resultados:**

Foram avaliados 56 pacientes no OG e 27 no GC. A média de idade foi de 36,4±8,5 anos. Os indivíduos do OG, em comparação com o CG, consumiram diariamente quantidades insuficientes de proteína e cálcio. O OG apresentou maior prevalência de baixa exposição solar, níveis mais baixos de 25OH vitamina D (21,3±10,9 vs 32,1±11,8 ng/ dL) e aumento dos níveis séricos de paratormônio (68,1±32,9 vs 39,9±11,9 pg/ml, p<0,001). Hiperparatiroidismo secundário foi presente apenas no OG (41,7%). A densidade mineral óssea da coluna lombar foi significativamente menor no OG. Quatro indivíduos do OG tiveram baixa densidade mineral óssea comparado com a idade cronológica, e nenhum do CG.

**Conclusão:**

Os componentes da dieta que afetam a massa óssea em pacientes submetidos à cirurgia bariátrica estavam inadequados. A suplementação alimentar foi insuficiente e a exposição solar baixa. Estas alterações foram acompanhadas de hiperparatireoidismo secundário e alta prevalência de baixa massa óssea em coluna lombar nestes pacientes.

## INTRODUCTION

Due to high rates of failure in clinical treatment of obesity, the number of surgical procedures for the treatment of this disease is growing exponentially. Bariatric surgery has an important therapeutic success in relation to weight loss, in addition to a substantial improvement in several co-morbidities^6^^,^ 2 ^,^ 27 ^,^ 30. The procedure however, can lead to significant nutritional deficiencies such as malnutrition, vitamin deficiencies, and changes in the metabolism of calcium and vitamin D. These changes are arising mainly from the low food intake and the intestinal malabsorption caused by the surgery^4^^,^ 18 ^,^ 29 ^,^ 32.

Several studies in the literature have shown a reduction in bone mass after weight reduction surgery^33^. The changes in fat mass and in the gastrointestinal tract, which occur after the surgical procedure, cause nutritional deprivations, change the mechanical strength of bone, interfere with the osteoblast differentiation and with the hormonal status, causing bone damage after bariatric surgery^12^.

The essential nutrients studied for the prevention and treatment of bone loss after bariatric surgery are protein, calcium, vitamin D, vitamin B12, and magnesium^34^.

The aim of this study was to evaluate some of these nutrients, linking food consumption with parameters of bone metabolism and serum concentrations of calcium, vitamin D, and parathyroid hormone. Furthermore, over one year after patients had undergone surgery by the gastric bypass technique, were evaluated the impact of the use of supplements and the sun exposure on their bone mass.

## METHODS

Was conducted an observational, cross-sectional, and single-center study at a private practice institute. The study was approved by Ethical Committee on Human Research, Hospital de Clínicas, Federal University of Paraná, Curitiba PR, Brazil. All patients signed an informed consent. 

Inclusion criteria consisted of men≥25 years, and women between 25 to 50 years with severe obesity, who had 12 months or more prior to participation undergone bariatric surgery by the Wittgrove bypass technique^35^ performed by one of the three surgeons participating in this study. 

Exclusion criteria were: patients unable to be contacted or who did not undergo the required exams; patients taking any drug or who had a disease that could interfere with bone metabolism; pregnant or menopausal women; women with any period of amenorrhea; and patients being treated for osteoporosis, except those taking calcium and vitamin D only.

The research protocol was conducted in three phases: the first one was a survey of the medical records of all patients who underwent the gastric bypass. Individuals who met the inclusion criteria were contacted by phone or mail and asked to participate in the second phase of the protocol. This phase consisted of a clinical interview regarding physical activity, sun exposure habits, the use of multivitamin and mineral supplements after the surgical procedure. In the third phase, blood and urine samples were collected in a single laboratory, previously defined by the study protocol and bone mineral density (BMD) was performed. 

The control group was composed of volunteers from family of the operated patients and was matched with the surgery group, according to sex, age, race, and BMI (Body Mass Index). For each control two operated were paired, with acceptance of age variations up to two years and up to two points in BMI. Individuals in the control group underwent the same evaluations as those in the operated group. 

### Assessment of nutritional status 

The patient's weight (kg), without shoes and wearing light clothing, was measured on a digital electronic balance. The height (meter) was measured by using a stadiometer. The BMI was classified according to the World Health Organization^21^. The weight before surgery was reported by the individuals and subsequently used to calculate the mean percentage of excess weight loss (%EWL)^5^.

### Food consumption evaluation

The 24-hour Recall and Food Consumption Semi-Quantitative Frequency questionnaire was used to assess dietary intake^11^. The 24-hour recall survey is based on the amount of foods consumed the day before the interview. It is the method of choice to estimate the absolute intake of energy and nutrients compared to a specific dietary recommendation. The Food Intake Frequency Inquiry is a method that measures dietary intake over a long period and consists of two basic components: a list of foods and a sequence of answers regarding the frequency and quantity of consumption. This questionnaire was based primarily on the food sources of calcium, vitamin D, protein, sodium, and caffeine. The Food Intake Frequency Inquiry was used in order to investigate whether the informed consumption in the 24-hour recall survey was compatible with the food habits of each individual. If there was any inconsistency in the items reported in the 24-hour recall survey, such as absence in the Food Intake Frequency Inquiry in five or more days of the week, new recall was carried out. 

Dietwin(r) software that contains a high number of foods high in sodium as instant meals, industrialized food, spices or beverages already registered, calculated the total sodium in the preparations. When patients eat outside home, it was asked if there was an extra addition of salt, and the sachet with 1 g of salt, available in restaurants, was used as reference. For those patients who usually prepared their meals at home, was investigated the amount of kitchen salt used per month by the family and the amount consumed per person was calculated. 

Caffeine intake was calculated considering the consumption of caffeinated beverages like coffee, tea and soft drinks. It was also questioned about the infusion of coffee or tea, if they were drank with milk or not, and the total volume of each. The dilution was questioned only when used instant coffee. 

The dietary assessment was calculated using the software Dietwin(r) Clinician 2002. To assess the adequacy of calcium, vitamin D, and protein intake, was used the Recommended Daily Intake according to sex and age (Institute of Medicine, 2011) as a reference standard and compared to the control group^19^. 

### Sun exposure, medications and physical activity 

The daily exposure to the sun was done throughout a questionnaire not yet validated for Portuguese, but used in other paper^20^. Patients were classified as follows: low sun exposure (exposure less than three times a week for less than a 15-minute period, exposing the face and arms); high sun exposure (exposure at least five times a week for more than 30 min, exposing the face, arms, or chest); or average exposure (those who were between the two criteria). 

All patients received the advice to maintain continuous use of calcium and vitamin D supplements, just after the procedure. At the moment of the study the participants were categorized into users or non-users, regarding the use of current calcium and vitamin D supplements, and medications after surgery. 

The practice of programmed physical activity was classified according to the intensity and frequency of exercises: mild, less than three times per week with practice of 1 h at a time; moderate, three to five times a week, with practice at least 1 h per time; and intense, at least five times per week over 2 h at a time.

### Biochemical analysis

Participants underwent a blood sampling after 10 h fasting. The following tests were performed: total calcium (Normal range - NR: 8.8-11 mg/dl); phosphorus (NR: 2.7-4.5 mg/dl); magnesium (NR: 1.9-2.5 mg/dl); albumin (NR: 3.5-4.8 g/dl); total alkaline phosphatase (NR: 53-128 U/l for men aged between 20-50 years, 56-119 U/l for men above 50 years and 42-98 U/l for women between 20-50 years); and parathyroid hormone (NR: 11-67 pg/ml). Serum 25OH Vitamin D (25OHD) levels were measured by chemiluminescence method using the Liaison(r) commercial kit and were classified according to the Endocrine Society's latest guidelines for vitamin D levels: deficiency, below 20 ng/ml; insufficiency, between 21 and 29 mg/ml; and normal, up to 30 ng/ml^16^. All samples of vitamin D, of patients and controls, were collected in the winter and fall. 

### Bone mineral density evaluation and body composition

BMD was measured by dual energy X-ray absorptiometry, in a Lunar DPX NT(r) model at the Center for Innovative Therapies. The results were evaluated by a single enabled physician. 

The regions evaluated were total body, lumbar spine - average L1-L4, femoral neck, and total femur. The BMD results were expressed as g/cm² and also through scores as compared to reference values determined by the International Society for Clinical Densitometry^28^.

### Statistical analysis 

Data are presented as mean± SD, except otherwise specified. All analyses were performed using SPSS v.20.0. The variables selected for statistical analysis were initially submitted to the Shapiro-Wilk test and the Kolmogorov-Smirnov test, which verified the assumption of their symmetrical (normal) distribution. Symmetrical distribution of the variables is presented as mean±standard deviation, while the asymmetric variables are presented as median, minimum, and maximum values. For mean comparison the Kolmogorov-Smirnov test was used. Fisher's and chi-square tests were applied for categorical variables, p values below 0.05 were considered statistically significant. For correlation analysis the Pearson's and Spearman's coefficients were utilized, which evaluated the association between continuous variables with symmetric and asymmetric distribution, respectively. In addition, multiple linear regressions were performed. For all analyses two-tailed tests with a minimum significance level of 5% were used.

## RESULTS

### Characteristics of control and operated groups

The medical records of 366 patients were evaluated. Of these, 219 did not fit the inclusion criteria and were excluded (62, incompatible surgical technique; 81, discordant age; 59, contact impossibility and 17, time from surgery smaller than one year). Of the 147 individuals invited to participate in the study, 70 did not accept the invitation. Thus, 76 patients were interviewed. However, 20 of these did not attend the required complimentary exams. Fifty-six operated (OG) and 27 adults without intervention, who comprised the control group (CG), totalized the 83 participants.

The OG consisted of 47 females (two African descent) and nine men (all White). The average postoperative time was 33.3±15.8 months. The mean age was 36.4±8.5 years and mean BMI was 28.2±4.2 kg/m^2^. The CG consisted of 20 women (one African descent) and seven men (all White), with a mean age of 36.9±9.6 years and mean BMI of 27.2±4.2 kg/m². The body composition characteristics were not different between the two groups. The mean %EWL of the OG was 73.5%±19.8. Nine individuals (16%) lost less than 50% of the weight excess. There was a difference between the BMI before (41.8±4.7 kg/m^2^) and after the surgery (28.2±4.2 kg/m^2^, p<0.001).

### Food consumption evaluation 

The average energy consumption in the OG was 1409.4±556.6 kcal/day, significantly lower compared to that in the CG, 2111.6±572.3 kcal/day (p<0.001). It was found that the amount of protein consumed (59.7±22.2 g/day in the OG versus 76.6±21.7 g/day in the CG, p<0.001) and the number of individuals consuming inadequate amounts of this macronutrient were different between groups 27 (48.2%) in the OG and only one in the CG (1.8%, p<0.001). The sodium intake was also lower in the OG 2425.1±958.8 mg/day vs 3651.2±998.4 mg/day in the CG (p<0.001). The calcium/protein ratio was inadequate in both groups (OG 8.1/1±3.7 mg/g vs CG 9.4/1±4.4 mg/g, Table 1).

**TABLE 1:** - Daily food consumption evaluation in the operated and control groups

**Variables**	**OG**	**CG**	**p**
TE (Kcal/d)	1409.4+ 556.6	2111.6 + 572.3	<0.01
Proteins (g)	59.7 + 22.2	76.6 + 21.7	<0.01
Proteins (g/kg/d)	0.8 + 0.3	1.02 + 0.2	NS
Calcium (mg)	486.8 ± 255.5	682.2 + 258.5	<0.05
Calcium/Protein (mg/g)	8.1/1 + 3.7/1	9.4/1 + 4.4/1	NS
Vitamin D (mcg)	2.4 ± 2.0	3.0 + 2.3	NS
Caffeine (mg)	231.4 + 195.2	255.8+ 139.1	NS
Sodium(mg)	2425.+ 958.8	3651.2 + 998.4	<0.01

OG=operated group; CG=control group; TE=total energy; NS=not significant

The daily dietary intake of calcium was lower in the OG than in the CG (486.8±255.5 mg/day vs. 682.2±258.5 mg/day, p<0.05). The number of individuals with a low dietary intake of calcium was higher in the OG (50 subjects - 91% - versus 20 subjects - 74% - in the CG, p<0.01). After surgery, 38 patients (67.8%) of the OG, used an average of 188.02±135.7 mg daily calcium supplementation for a mean period of 16.1 ±11 months. In the operated individuals who used supplementation, calcium intake became similar to the CG (615.97±308.2 mg/day).

**FIGURE 1:**
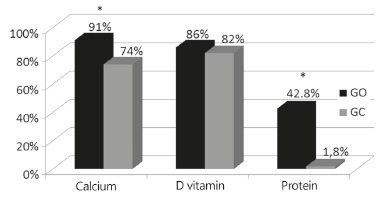
- Prevalence of inadequate dietary intake of calcium, vitamin D, and protein in the operated and control groups

The number of individuals with an inadequate intake of vitamin D was similar between groups: OG, 47 patients (86%); and CG, 22 patients (82%). The dietary intake of vitamin D was similar between groups (OG 2.4±2.0 mg/day or 96±80 IU/day vs CG 3.0 ±2.3 mg/day or 120±92 IU/day). The same 38 subjects who were taking calcium supplementation in OG also were taking vitamin D supplementation on an average of 10.54±1.35 mcg/day or 423.7±54.2 IU for a mean period of 16.1±11 months, bringing the total average intake (diet + supplementation) to 12.94±3.35 mcg/day or 517.6±134 IU/day. A summary of the percentage of patients who had inadequate nutrient intake in both groups is shown in Figure 1.

OG=operated group; CG=control group;*=p<0.01

Sun exposure and physical activity 

**FIGURE 2:**
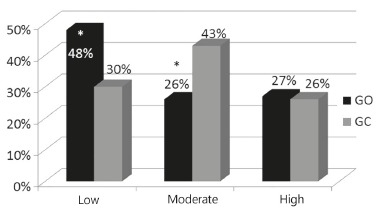
- Prevalence of low, moderate, and high sun exposure in operated and control groups

The prevalence of individuals with high sun exposure was similar between the two groups (OG 27% vs CG 26%). However, the prevalence of low sun exposure was higher in the OG when compared to the CG (48% vs 30% respectively, p<0.01), whereas the CG showed a higher prevalence of moderate exposure (43% vs 26%, p<0.05, Figure 2).

OG=operated group; CG=control group;*=p<0.01

 Regarding the physical activity, 36 (64.3%) subjects were classified as sedentary, 14 (25 %) as mildly physically active and six (10.7%) as moderately active. 

### Biochemical analysis

The mean serum levels of calcium, magnesium, and phosphorus were within normal values without difference between groups. The OG showed higher levels of phosphorus (3.8+0.5 mg/dl) than the control group (3.1+0.5 mg/dl, p<0.001), lower levels of 25OHD (21.3±10.9 vs 32.1±11.8 ng/dl, p<0.01), and increased serum levels of parathyroid hormone (68.1±32.9 vs 39.9±11.9 pg/ml, p<0.001). 

Secondary hyperparathyroidism, characterized by elevated levels of parathyroid hormone (>67 pg/ml), low vitamin D (<30 ng/ml) and low or normal calcium, was present in 20 patients in the OG (41.7%) and in none of the CG. Deficiency of vitamin D was observed in 28 patients (58.5%) in the OG and in three (16.6%) in the CG (p<0.001). Insufficiency of vitamin D was similar between the groups, 12 (25%) in the OG and 6 (33.3%) in the CG.

The linear correlation analysis showed an inverse correlation between 25OHD and parathyroid hormone levels in the OG (r= -0.41, p=0.005) and in the CG (r= -0.61, p=0 01). No correlation was found between the 25OHD levels and the intake of vitamin D, the use of supplements, BMI, fat mass, lean mass, %EWL, and sun exposure. Multiple linear regression analysis showed that serum calcium, BMI, %EWL, and parathyroid hormone together explained 65% of the alterations in 25OHD levels.

### Bone mineral density evaluation

The BMD in total body, total femur, and femoral neck were similar between groups, but the mean BMD in lumbar spine was lower in the OG, 1.203±0.15, compared to the CG, 1.282±0.16, p<0.05). Four individuals from the OG (8%) had low BMD for chronological age (Z-score <-2.0), and none from the CG (p<0.05). Osteoporosis was not observed in either group.

In the OG, the total body BMD was significantly lower in patients with secondary hyperparathyroidism, 1.161±0.068, compared to those without, 1.217±0.109 (p<0.05). No difference was found between BMD and the use of supplements of calcium and vitamin D in the OG. No correlation was found between the time after surgery with the levels of 25 OH D, parathyroid hormone, alkaline phosphatase, or BMD. In linear regression analysis, there was no correlation between BMD and physical activity. Multiple linear regression analysis showed that caffeine intake, levels of magnesium, alkaline phosphatase, and 25 OH D together explained 44% of the variations in lumbar spine Z-score in the OG. There was a positive correlation between the total body BMD with serum calcium (r=0.36; p=0.03), 25 OH D (r=0.37; p=0.01), BMI (r=0.27; p=0.05), lean mass (kg) (r=0.48; p=0.0005) and an inverse correlation with the %EWL (r= -0.29; p=0.04). The total body BMD was positive correlated with the lean mass (kg) (r=0.36; p=0.01) and negative correlated with the %EWL (r= -0.29; p=0.04). 

## DISCUSSION

Nutritional deprivation even previous surgery in obese patients^6^ and aggravated after surgery with the weight reduction, explaining the reduction seen in bone mass^33^. According to the latest recommendation from the Institute of Medicine, the correct amount of protein intake is 0.66 g/kg/day. In our study it was seen that 48.2% of the operated subjects consumed inadequate amounts of this nutrient. These data are similar to those^3^, which showed after one year of gastric Roux-en-Y procedure bypass, an average of daily protein intake of 0.5±0.3 g/kg/day. In another study that evaluated the same technique, 10,2% consumed insufficient amount of protein^31^. 

In the general population, the deficiency in calcium intake is evident. In a study of 2.420 Brazilians, it was seen that 99% of the population had inadequate intake, the average consumption was only 52% of daily needs^24^. This study confirmed these data; both groups were ingesting calcium below the recommended daily amount, and in the OG was still lower, even in the patients who were supplementing calcium, 615.97±308.2 mg/day. According to the latest recommendations of the Institute of Medicine, women and men aged 30-50 years should ingest 1.000 mg of calcium per day. Various studies have demonstrated the low intake of calcium after bariatric surgery^21^^,^ 31. Another important factor is that, besides the reduction in dietary intake of calcium, a reduction in their absorption of up to 34% after six months of the procedure occurs due to the changes in the anatomy of the gastro-intestinal tract^26^. 

In this study, caffeine consumption was not considered high and was similar between the two groups, even though some studies had associated the high caffeine intake with reduced bone mass in individuals with other risk factors^13^. However, the data regarding post-bariatric surgery are scarce.

As calcium intake, levels of vitamin D intake were far below the recommended by more than 80% of patients in both groups. This low consumption occurred even in patients who were receiving supplementation (517.6±134 IU/day). The daily recommendation of vitamin D intake by Institute of Medicine is 600 IU/day for individuals below 70 years. Several studies have also found a low intake of this vitamin^17^, reaching 95% of patients with an intake below the recommended post-surgery^17^. The lack of food fortification is most likely responsible for this low intake in both groups, as enriched foods with vitamin D are not part of the usual diet in Brazil.

Was found a high prevalence of secondary hyperparathyroidism in the OG (41.7%), confirmed by the literature data. In 125 patients operated by the same surgical technique, 40% had secondary hyperparathyroidism^15^. The vitamin D deficiency and secondary hyperparathyroidism is commonly observed in obese individuals and may be aggravated after weight loss induced by bariatric surgery. Probably, the pre-existing factors such as inadequate food intake and insufficient sun exposure were aggravated during the postoperative period by malabsorption of calcium and vitamin D^14^. While the prevalence of hyperparathyroidism after a variety of bariatric operations is well known, this does not clearly relate directly to vitamin D or calcium intake or meaningful deficiency in some studies^25^. Presence of hypocalcemia was seen in 14.3% of patients, which is consistent with the literature, since most patients with secondary hyperparathyroidism have normal serum calcium^22^. In 110 patients who underwent surgery by Capella technique, 29% had high PTH but only one had hypocalcaemia^9^. 

The vitamin D deficiency was observed in 58.5% of the OG and insufficiency in 25.3%, similar to a study that showed similar results three years after gastroplasty, 43.2% and 34.1%, respectively^36^. If was used the Institute of Medicine criteria, the prevalence of hypovitaminosis D would have been lower in the OG (44%), but still high.

No correlation was observed between 25OHD levels and vitamin D intake, use of supplements, and BMI. Although sun exposure was low in 43% of the OG, no correlation was observed with the 25OHD levels. One of the probable reasons for this lack of association was the missing analysis of skin color subtype, considering that each skin type needs a specific solar exposure time for adequate vitamin D production^8^. Another likely reason for the deficiency would be the sequestration of vitamin D in adipose tissue of the obese patients, and not a reduction in sun exposure^1^. 

This study showed a low bone mass, at least in one site, for chronological age in 8% in OG, which was not observed in the CG but was similar to the data in the literature^10^. 

Calcium and vitamin D supplementation was inadequate in this study; most patients used multivitamins and polyminerals available over-the-counter as a form of general supplementation. After bariatric surgery the supplementation should be considered a medical prescription, and individualization is also required. 

Another restricting factor is the difficulty in longer follow-ups with bariatric patients, limiting the establishment of studies and protocols for this group of patients.

## CONCLUSION

The dietary components, protein, calcium, and vitamin D, which affect bone mass in patients undergoing bariatric surgery, were inadequate, as well as the sun exposure. These changes were accompanied by secondary hyperparathyroidism and a high prevalence of low bone mass in lumbar spine. Thus, should not only alert the medical staff but also patients for these possible alterations, and encourage adequate dietary intake of each nutrient, explaining the importance of proper supplementation.
